# “Withstanding ambivalence is of particular importance”—Controversies among experts on dealing with desire to die in palliative care

**DOI:** 10.1371/journal.pone.0257382

**Published:** 2021-09-24

**Authors:** Kerstin Kremeike, Thomas Dojan, Carolin Rosendahl, Saskia Jünger, Vanessa Romotzky, Kathleen Boström, Gerrit Frerich, Raymond Voltz

**Affiliations:** 1 Department of Palliative Medicine, Medical Faculty, University of Cologne, Cologne, Germany; 2 Department of Community Health, University of Applied Health Sciences, Bochum, Germany; 3 Center for Integrated Oncology Cologne / Bonn (CIO), University of Cologne, Cologne, Germany; 4 Center for Health Services Research Cologne (ZVFK), University of Cologne, Cologne, Germany; University of Auckland, NEW ZEALAND

## Abstract

In order to investigate controversies surrounding the desire to die phenomenon in palliative care by analyzing expert opinions on the topic, we carried out a secondary qualitative data analysis of free text comments collected during a Delphi survey that was designed to develop a conversation aid for dealing with desire to die in everyday clinical practice. Between 01/2018 and 03/2018, a two-round Delphi survey was carried out with national (German) and international palliative care experts. Free text comments were reinvestigated to identify controversies surrounding the desire to die phenomenon. An additional in-depth analysis focused on statements expressing attitudes towards proactively addressing (potential) desires to die. Within the Delphi survey, 103 of 149 multi-professional participants (almost all of them with practical and only six with exclusively theoretical expertise in palliative care) generated 444 free text comments. Thereof, we identified three main categories related to dealing with desire to die: “outer framework“, “extended care system” and “health-professional-patient-relationship”. Ambivalences, taboos and uncertainties surrounding desire to die in palliative care became apparent. Experts are divided concerning the practice of proactively addressing desire to die. Even if these conversations–especially the proactive approach–are also viewed critically, we conclude that open-ended and respectful communication about desire to die between health professionals and patients can be understood as an eligible intervention in palliative care. Proactively addressing the topic is a possible way to open up such conversations.

## Introduction

According to the WHO-definition, „palliative care is an approach that improves the quality of life of patients and their families facing the problems associated with life-threatening illness, through the prevention and relief of suffering by means of early identification and impeccable assessment and treatment of pain and other problems, physical, psychosocial and spiritual” [1]. In palliative care, most patients are likely to think about the end of their lives at some point during their disease trajectory. Not few of these patients experience desires to die in various forms, intensities and functions [2]. Desire to die can occur with a simultaneous will to live, with both being prone to change over time. The resulting complexity of the desire to die phenomenon, as well as uncertainty concerning the right way to deal with it, can lead health professionals to neglect or avoid the issue. Some health professionals believe that patients will initiate conversations on their own should they wish to talk about the end of life and desire to die; they worry about the potential adverse effects of bringing up the topic in conversations themselves [3]. Opposing opinions value the potentially positive effects of desire to die discussions for patients such as reduced depressive symptoms [4].

Bringing together the different viewpoints on this topic and focusing on controversies surrounding the proper practice when dealing with desire to die in palliative care could provide health professionals with a more nuanced and extensive view on the topic.

Desires to die understood as a phenomenon with various forms, intensities and functions occur frequently in palliative care [5]. Studies report the occasional experience of desire to die in up to 45% of patients with advanced cancer; up to 12% show a strong and persistent desire to die [6, 7].

Different approaches exist to define desire to die. An international consensus definition describes the ‘wish to hasten death’ as a reaction to suffering from which the patient can see no way out, except to accelerate death [8]. Therewith, the wish to hasten death is distinguished from the acceptance of impending death or the wish to die naturally. Using the term desire to die, we go beyond this narrow definition and understand the phenomenon as a broader concept [5]. Besides a wish to hasten death even without requiring any accelerating action, a request for assisted dying and suicidal ideation, desire to die may–from this point of view–be expressed as the acceptance of death or satiety with life. Desire to die may also lead to the construction of a specific exit-plan in case of further physical or psychological deterioration [9]. This understanding is in line with the *German Palliative Care guideline for patients with incurable cancer* which conceptualizes desire to die along a line of increasing pressure to act [5, 10]. The will to live is described as a similarly complex phenomenon which can persist throughout the entire dying process even if it is permeated by experiences such as hopelessness, the feeling of being a burden to others and loss of dignity [7].

Whereas desire to die is a subjectively experienced phenomenon and therefore requires special attention to the affected patient as an individual, its potential realization as euthanasia or assisted suicide depends on the legal context of each country. Our study was conducted among mostly German health professionals and at a time during which euthanasia ("termination of life on request"; § 216 national criminal code) was forbidden and assisted suicide ("assistance of suicide with intent of repeated conduct”; § 217 national criminal code) was legally restricted in Germany [11, 12]. However, §217 has been repealed in 2020 [13]. A recently published review investigated the discussion on termination of life on request in Europe during the last two decades [14]. The example of the Netherlands shows that legislation of termination of life on request goes hand in hand with an increase of cases administered. An analogous (but small) increase in other European countries that have legislated euthanasia (e.g., Belgium) can be seen as well. Another trend seems to be that the originally strict applicability of safety provisions (e.g., ‘suffering hopelessly and unbearably’) are with time gradually expanded such that less serious conditions (e.g., life fatigue in old age) are sufficient for the administration of termination of life on request. In order not to turn termination of life on request into a standard procedure in end of life care, the relationship between suicide and suicide prevention on the one hand and euthanasia acts and promotion of euthanasia on the other is of great importance. The role of palliative care in societies’ approach to end-of-life care is seen as crucial in this regard.

The complexity of desire to die causes uncertainty in health professionals on how to deal with the issue [15]. Therefore, a first needs-oriented training program for dealing with desire to die was developed and a clinical approach on addressing desire to die was drafted [16]. This clinical approach has been further developed and consented, *inter alia* by a Delphi survey with professional experts [17]. While experts reached consensus on the design of the clinical approach for everyday practice, they voiced divergent opinions in free text comments on how to best deal with desire to die. These free text comments were reinvestigated by secondary data analysis.

Even though this work predominately addresses the context provided by the German legal framework, we believe that our findings hold value for an international audience as the topic is also discussed across national borders. By studying desire to die, we address an existential issue relating to the human condition as such.

### Aims

The study aimed at investigating controversies surrounding the desire to die phenomenon in palliative care by analyzing expert opinions. This is meant to build awareness for different viewpoints, to support health professionals in reflecting their own attitudes, and to derive practical conclusions concerning the handling of desire to die. Our research addressed the question: What controversies surrounding dealing with desire to die do experts in the field currently discuss?

## Materials and methods

### Design

We report a secondary analysis of qualitative data generated during a two-round Delphi survey. The survey aimed at building consensus on the content and structure of the ‘semi-structured clinical approach on (proactively) addressing desire to die’ in patients receiving palliative care [17]. It was part of a three-phase research project aiming at optimizing the handling of desire to die in palliative care [18]. The Delphi technique is a method of collecting opinions on research questions in fields of relative uncertainty with both quantitative and qualitative methods [19]. The Delphi survey was carried out applying the guidelines for conducting and reporting Delphi Studies (CREDES) in palliative care [20]. Participants rated statements on multiple domains of the clinical approach draft that was based on focus groups, literature research, advisory board recommendations and patient interviews [16, 17]. The survey included the opportunity to provide free text comments. These comments were reinvestigated applying qualitative content analysis [21, 22] to categorize controversies surrounding desire to die. An additional in-depth analysis focused on statements related to proactively addressing desire to die. We report our study in accordance to the synthesis of recommendations for reporting qualitative research [23].

### Sample

**Fig 1:**
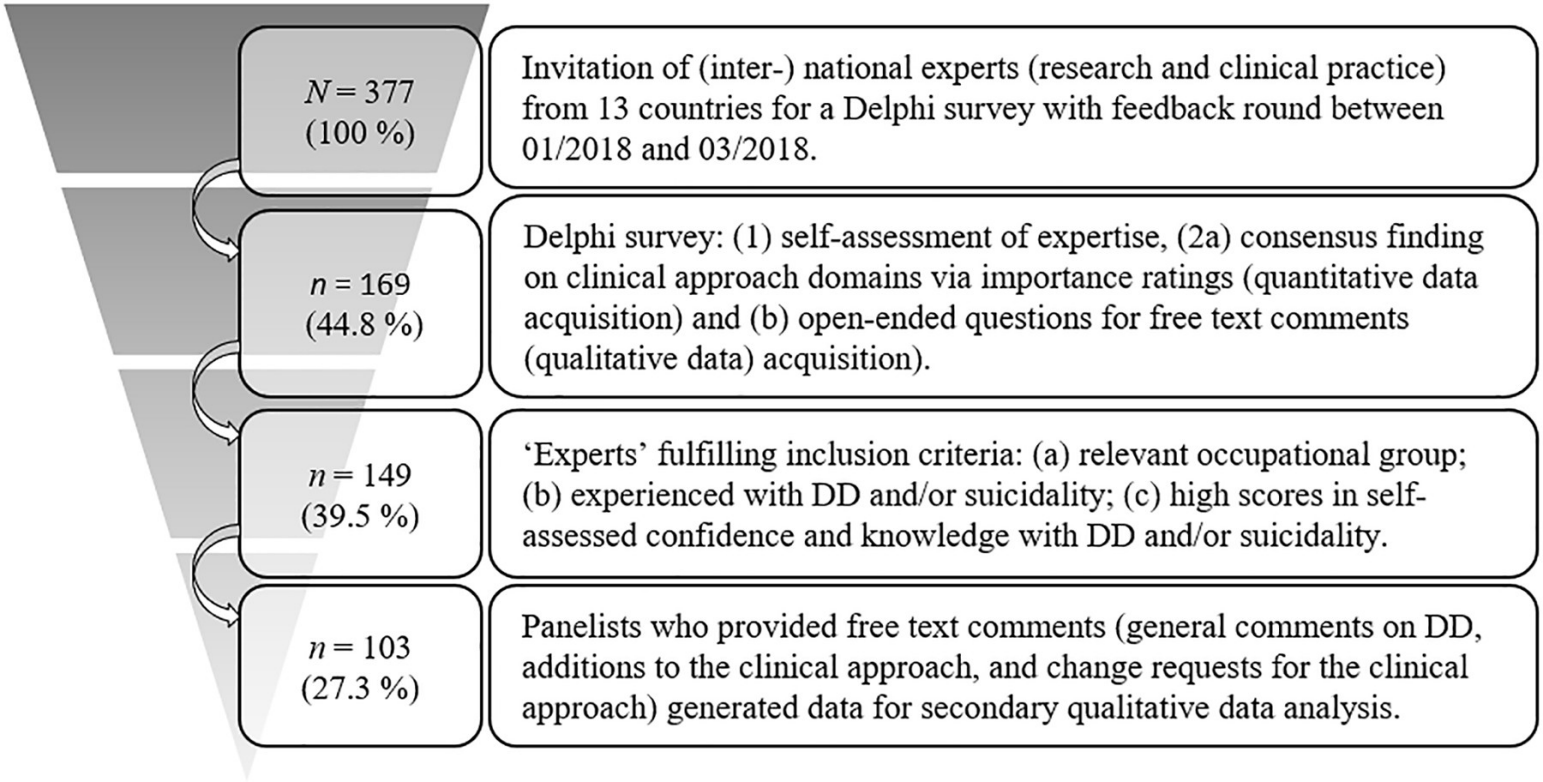
Research sample extraction.

In our Delphi survey, we addressed palliative care experts in order to develop a semi-structured clinical approach on dealing with desire to die [18]. We aimed at recruiting a heterogeneous sample of practitioners and theoreticians as both groups are involved in the international debate concerning desire to die in palliative care [17]. The sample for secondary data analysis was extracted from the original survey sample by singling out all respondents who met the inclusion criteria (‘experts’) and provided free text comments (see Fig 1). We thus identified 149 experts among our participants–a significantly higher number of participations as recommended as minimum according to the CREDES recommendations (50 to 70) [20] and as originally aimed at [18]. A detailed description of the source sample can be found in an earlier publication [17]. A subsample of 103 panelists was identified for qualitative data analysis of 444 free text comments stated during the Delphi survey. This equates 69% of all panelists fulfilling inclusion criteria (*n* = 149). Of the 444 comments, 330 were considered substantial. 114 comments that did not contain data fit for qualitative text analysis (e.g. brief comments such as “yes” or “good”) were excluded from analysis.

### Data collection

The online Delphi survey was carried out between 01/2018 and 03/2018. Participants were asked to give free text comments on eleven domains of the clinical approach (see Table 1). They were instructed to suggest further recommendations, request changes or simply state their opinion freely. For a complete description of the clinical approach and its development, please refer to Kremeike, Frerich (17).

**Table 1:** Eleven domains of the semi-structured clinical approach as originally presented in Kremeike, 2020 [14].

**A–Usage Notes**
1. Usage notes
**B–Conversation Aspects**
2. Actively building the relationship
3. Proactively addressing desire to die
4. Closure of discussion
5. After discussion
**C–Classification, Meaning and Functions**
6. Classification of desire to die
7. Background and meanings of desire to die
8. Functions of desire to die
**D–(Self-)Reflection**
9. Conscious engagement with own attitudes and emotions
10. Self-protection
**E–Further Recommended Action**
11. Further recommended action

### Ethical considerations

Research was conducted in accordance with the Declaration of Helsinki. Ethical approval for this study was obtained from the Ethics Committee of the University of Cologne (#17–265). All participants signed a declaration of consent.

### Data analysis

Free text comments were analysed by three data coders (TD, KK, CR) utilizing the qualitative data analysis software MAXQDA 18 [24]. Applying qualitative content analysis, we identified and systematized inductive thematic categories [21, 22].

While ten domains of the semi-structured clinical approach on dealing with desire to die reached an agreement of ≥ 90% among the 149 Delphi panelists, the domain on “proactively addressing desire to die” received only 83.2% of agreement [17]. This domain was also commented on most extensively. Apparently reflecting a self-contained controversy, related comments were found suitable for evaluative qualitative content analysis. Thereof we extracted an ordinal agreement-disagreement scaling.

#### Rigour

Throughout the entire coding process a cooperative consensual coding approach [21] was applied, ensuring inter coder reliability by constant comparison between coders and according adjustments of the category system.

## Results

The analyzed Delphi subsample consists of about two thirds of women and one third of men aged 19 to 69 years. Panelists were able to give multiple responses on their expertise as this mirrors their multi-professional profiles–only six of our panelists reported exclusively theoretical expertise (e.g. researchers with no practical experience)–thus presenting a panel of mostly practical professional experts. All sociodemographic data was self-reported by the panelists. For more details, see Table 2. In comparison with the source sample (*N* = 149 experts), the subsample of *N* = 103 commentators shows no notable differences concerning any of the sociodemographic characteristics (for details see Table 2 in our earlier publication [17]).

**Table 2:** Sociodemographic data of commentators.

Commentators, *N* (%)			103 (100.0)
Age, *M* (*SD*)			50.0 (9.0)
Gender,^a^ *n* (%)	Male		32 (31.1)
	Female		71 (68.9)
Residence, *n* (%)	Germany		87 (84.5)
	Other countries		16 (15.5)
Expertise,^b^ *n* (%)	Nursing		57 (55.3)
	Physician		17 (16.5)
	Research and science		17 (16.5)
	Non-practitioners, e.g. moral philosophers		12 (11.7)
	Ethics counseling		9 (8.7)
	Relatives		6 (5,8)
	Psychology and psychotherapy		8 (7.8)
	Spiritual care		8 (7.8)
	Other		13 (12.6)
Experience in years, *n* (%)	Dealing with desire to die	≤1	2 (1.9)
		1–9	35 (34.0)
		≥ 10	63 (61.1)
	Dealing with suicidality	≤1	27 (26.2)
		1–9	26 (25.2)
		≥ 10	47 (45.6)
	Desire to die theory	≤1	34 (33.0)
		1–9	45 (43.7)
		≥ 10	17 (16.5)
	Suicidality theory	≤1	52 (50.5)
		1–9	29 (28.2)
		≥ 10	15 (14.6)
Confidence,^c^ *M* (*SD*)	Dealing with desire to die		4.2 (1.0)
	Dealing with suicidality		3.1 (1.3)
Knowledge,^c^ *M* (*SD*)	Desire to die		4.1 (1.0)
	Suicidality		3.0 (1.4)

^a^Gender was self-reported between the options ‘male’, ‘female’ and ‘other’ (with an option to specify in free-text). However, the participants did not use the category ‘other’.

^b^Multiple responses possible.

^c^0 to 6 Likert scale.

### Thematic categories

**Fig 2:**
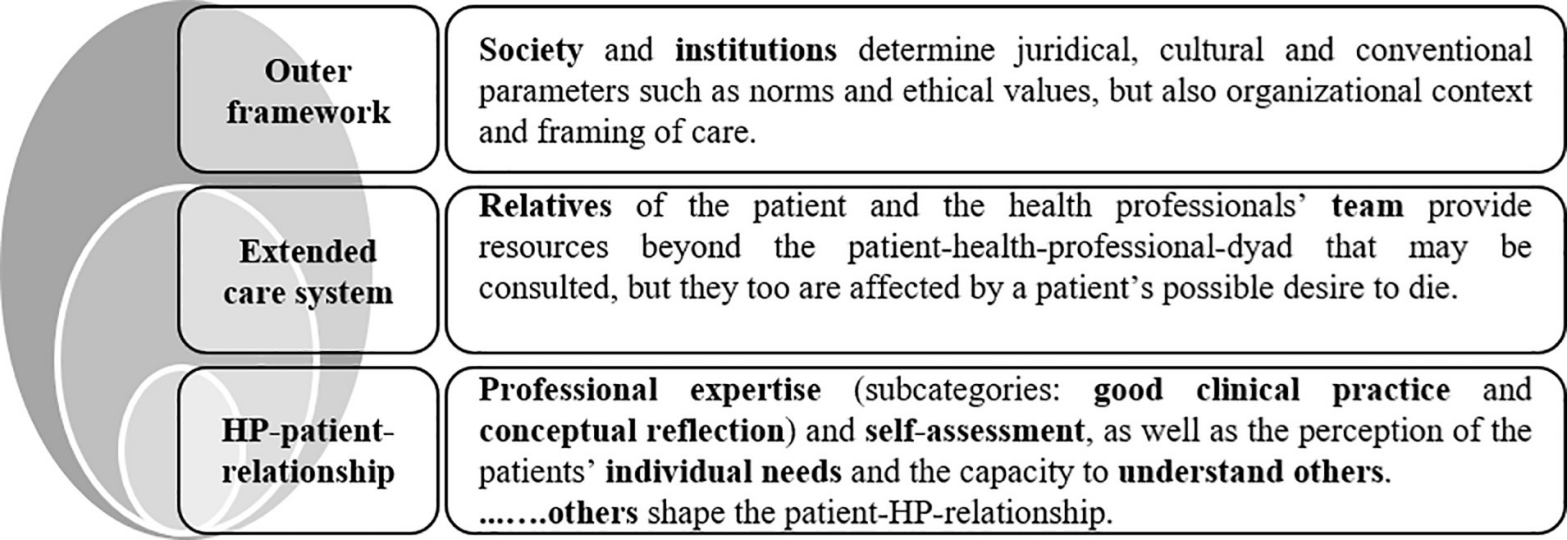
Thematic categories describing structures with an impact on dealing with desire to die.

Three thematic categories were identified for the free text comments on all domains of the clinical approach. They revolve around structures with an impact on dealing with desire to die: “*outer framework*”(*n* = 58 comments), “*extended care system*” (*n* = 28 comments) and “*health-professional-patient-relationship*” (*n* = 244 comments). Panelists voiced opinions about how each of these structures influences dealing with desire to die in palliative care and conversations (in general and about desire to die in particular) between health professionals and patients. Fig 2 displays the categories’ descriptions.

#### Outer framework

On this level, we differentiated comments concerning the influence of *society* (*n* = 17) and *institutions* (*n* = 41) on dealing with desire to die in palliative care.

*Society* includes all comments on juridical aspects of palliative care, cultural conventions and societal attitudes towards death, dying and end of life care (specifically on ‘assisted suicide’), norms and ethical values. Comments highlight the relevance of desire to die but also reflect the insufficient definitions of and distinction between ‘desire to die’, ‘assisted suicide’ and ‘euthanasia’ in public discourse. This may lead to patients and their relatives (as well as health professionals) tabooing the topic:

*”Having in mind that people who come to us might not have any earlier experiences with end of life*, *death and dying; thus also the vocabulary might be lacking and the entire topic might be perceived as taboo*.*”*(Clinical psychologist, #0193)

In order to have a patient-centered conversation about desire to die, respondents stress the importance of knowing in which way to talk about it. Depending on patients’ socio-cultural background, norms and values, there may be different ideas of appropriateness and ethical convictions ranging from openness to taboo:

*”It is a topic where not only personal perspectives play a role*, *but also family*, *belief*, *culture*, *milieu and one’s own history*.*”*(Palliative care nurse, #356)

Comments on palliative care in general (not on the health-professional-patient-interaction in particular) such as the organization and framing of palliative care (e.g. setting) are grouped within the category *institution*. Institutional options can have an impact on the provision of a certain intervention such as medical or psychological therapies. Ensuring best practice not only depends on the general existence of specific treatment and care services, but also on their availability on short call:

*“Intervention options must also provide the possibility of prompt implementation*. *Otherwise*, *people in distress cannot do anything with it and may even feel more desperate*. *Psychotherapies*, *for example*, *require a long waiting period*.*”* (Nurse and ethics counselor, #367)

The importance of discussing possible modifications of therapeutic goals was stressed in order to best deal with desire to die and alleviate existential despair:

*“When dealing with desire to die*, *we should primarily talk about options for treatment continuation and discontinuation*. *Else we drive people into suicide because we do not inform them about discontinuation [of treatment]*, *[palliative] sedation etc*.*”* (Physician, #0131)

#### Extended care system

The extended care system has an impact on dealing with desire to die in the way *relatives* (*n* = 8 comments) and the *team* (*n* = 20 comments) are affected.

*Relatives* are involved in looking after patients and too are affected by patients’ distress. They also provide resources for the provision of optimal care by health professionals. Thus, relatives should be involved in conversations about desire to die:

*“[Consider] offering opportunities to resume the conversation with […] family*, *friends*, *[…]… depending on individual triggers for expressed desire to die*.*”* (Hospice care coordinator, #0146)

All free texts that point towards the *team* as a resource for relief and reflection are grouped together. They suggest that interdisciplinary work enables the best possible support of those patients that wish to die:

*“Sharing information with the multi-professional team […] and delegating tasks with assisting professions is hugely important*.*”* (Elderly care trainer. #354)

However, discussing a patient’s desire to die with the team poses challenges as well, especially when no intervention seems immediately warranted:

*“Withstanding ambivalences is of particular importance*. *Palliative care teams*, *too*, *seek to reason everything out*, *but that is not always possible in these challenging situations*.*”* (Physician, #0141)

Uncertainties or dissent can arise among team members about how to deal with a particular patient’s desire to die. Ethical advice was pointed out as helpful to find approaches that are morally sound for all involved:

*“An ethical case conference might be convoked if the health professional caring for a patient with desire to die (or their team) perceive the need to do so; e*.*g*., *when a patient’s desire to die overwhelms some team members and they wish to have collegial exchange*.*”* (Neurologist, #0186)

#### Health-professional-patient-relationship

The largest number of comments (*n* = 244) related directly to the health-professional-patient-relationship. It subdivides into health professionals’ abilities in *understanding others* (*n* = 36), their attunement towards a patient’s *individual needs* (*n* = 55), the health professionals’ *professional expertise* (*n* = 120) and their *self-assessment* (*n* = 33).

*Understanding others*. The importance of acknowledging divergent experiences (e.g. wanting to live vs. having a desire to die) and conflicting values (e.g. the protection of the sanctity of life vs. respect for patients’ autonomy) was stressed:

*“[Health professionals should] accept that oneself and one’s own needs may disappear for a while during somebody else’s dying process*.*”* (Volunteer, #0118)

*Individual needs*. Our panelists voiced concerns that the individual needs of a patient can–especially in the desire to die context–neither be reduced to ‘business as usual’ approaches, nor can they be adequately subsumed in broad theories about desire to die:

*“Most important for [the clinical approach] is to stress that each patient is to be understood within their individual biography and that this cannot be generalized to others*.*”* (Palliative care ward manager, #373)

*Professional expertise*. The two subcategories *good clinical practice* (*n* = 85 comments) and *conceptual reflections* (*n* = 35 comments) have been discerned on this level.

***Good clinical practice.*** This subcategory includes all comments about communicational skills, health professionals’ duties as caretakers, individual (in)securities and keeping up with state-of-the-art practices related to desire to die. Building and maintaining relationships with the patient and knowing which interventions are adequate were highlighted as important. Open-ended communication should be understood as an appropriate action in this context, often establishing the basis for patients to talk about their desire to die at some point:

*“Problem solving is the foremost reason for initiating conversations in everyday clinical practice*. *[…] However*, *communication that allows (and recommends) participants to talk with each other for the sake of communication–open-ended*, *free of specific goals and documentable results–is an option*, *too*. *[…] Health professionals should understand that these are professional conversations as well*.*”* (Hospice care coordinator, #0146)

***Conceptual reflection.*** All comments on theoretical and practical knowledge regarding desire to die, suicidality, mourning and depression as well as the need for differential diagnostics are included in this subcategory. The most common themes were the complexity and changeability of desire to die. In addition, the ambivalence between the will to live and desire to die was referred to:

*“Wish to hasten death should not overshadow the wish to live*… *not to give up; both desires can be found in the same person simultaneously*.*”*(Palliative care researcher, #0136)

Besides symptom control, the focus in palliative care is on dealing openly and dignified with the patients’ beliefs, wishes and burdens. The health professional should not try to talk their patient out of their desire to die, but try to understand it as best as possible.

*“A palliative focus should be less concentrated on the avoidance of suicide*. *On the one hand we should aim at identifying and alleviating pain while on the other hand accompanying patients in their suffering as dignified persons towards the end”*. (Palliative care nurse, #382)

*Health professionals’ self-assessment*. In order to facilitate long-term relationships and open communication about existential issues, health professionals should also accept their own limits. Here, the relevance of reflecting one’s own (emotional) experiences, (ethical) values and self-care was emphasized.

*“Everybody has different limits*. *Authentic companionship includes the freedom to draw a line if a situation overwhelms me*.*”* (Companion for the dying, #0203)

**Fig 3:**
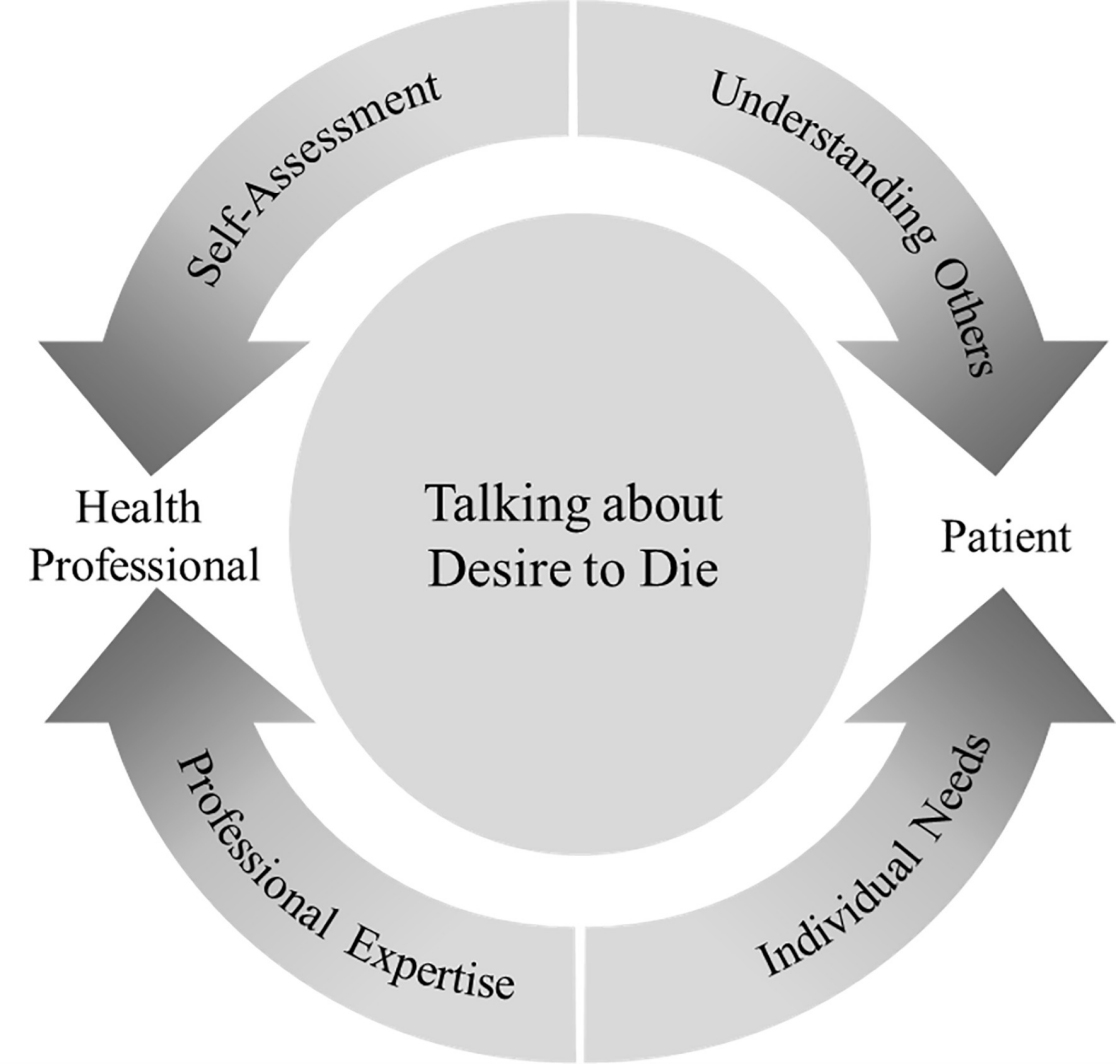
Tensions and ambivalences in the health-professional -patient-relationship as related to desire to die conversations.

Within the various aspects of the health professional-patient-relationship, tensions and ambivalences emerge (see Fig 3). Health professionals and their patients are two participants of the care relationship and any communication therein. Therefore, health professionals must be able to alternate between focusing on their own viewpoints during self-assessment while also being able to take the patient’s perspective. Confronted with a patients’ desire to die, health professionals are also in charge of arranging the proper balance between their professional expertise regarding the phenomenon in general and the attunement to individual needs, which may differ from case to case.

#### Evaluative category on “Proactively addressing desire to die”

**Fig 4:**
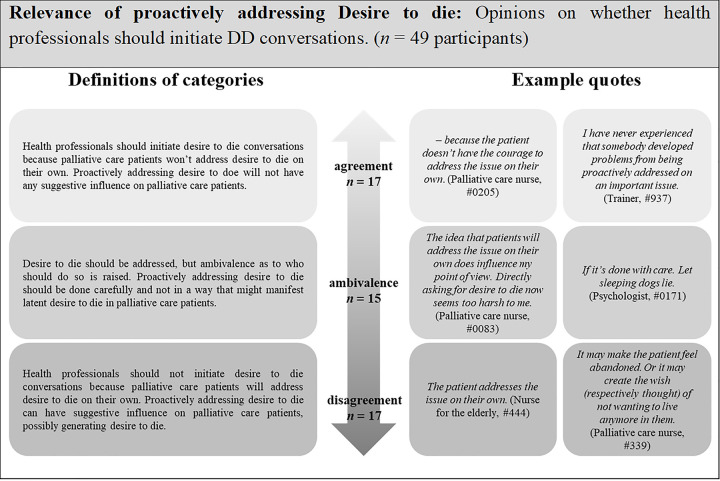
Evaluative category related to ‘proactively addressing desire to die’.

Among the 244 comments on the health professional-patient-relationship, 49 free texts expressed varying degrees of agreement with the recommendation that health professionals should proactively address desire to die with patients in palliative care (see Fig 4). There were no significant differences in opinion between subgroups (such as different professions) of the source sample [17]. The socio-demographic data of this sub-group largely mirrors the distributions of the overall sample (see Table 2): female and male participants are distributed roughly with a two to one ratio (71.4% female, 28.6% male), residence states are Germany (81.6%) and other countries (19.4%) and the mean age is *M* = 50.8 (*SD* = 8.6). The distribution of expertise among participants is, again, the same: the panelists are multi-professional practitioners, e.g. reporting 61.2% ‘nursing’ and 12.2% ‘research and science’ among other options on the multiple response format. We therefore conclude that no systematic selection took place via the option to comment on ‘proactively addressing desire to die’.

The group of commentators agreeing that health professionals should proactively address desire to die with patients (*n* = 17 participants) believe that this is part of the assignments of palliative care and they do not think that a proactive approach poses an iatrogenic risk (i.e. has detrimental suggestive influences).

The ambivalent commentators (*n* = 15 participants) rate speaking about desire to die as important, but wish that it only takes place when there are indicators or when an adequate situation arises. They fear that a proactive approach may overstep boundaries or manifest latent desire to die in patients.

The disagreement group (*n* = 17 participants) oppose proactively addressing desire to die because they believe that patients will talk about their desire to die on their own should they want to do so. Some believe in a suggestive influence of a proactive approach on patients to develop a desire to die. Others believe that health professionals are not skilled enough in conversation techniques to be able to address the sensitive issue adequately.

#### Effects of age, expertise and gender

No effects of age or expertise were found across both, thematic and evaluative categories. For all evaluative and most thematic categories, no effects of gender were found either. That is to say, that the distribution between the genders of the panelists that stated the comments included in a given category mirror the gender distribution of the overall sample (see Table 2), i.e. they have or are close to a two to one ratio of female vs male gender. However, three codes deviated notably from this general descriptive trend: 18.4% more women commented on the importance of ‘individual needs’, 18.6% more women commented on the role of ‘relatives’ and 16.0% more men commented on the influence of ‘society’.

## Discussion

Health care for patients receiving palliative care is carried out in many different institutions [25], resulting in complex interactions between agents operating on different levels of societal systems. Data collected from our Delphi panelists revealed these levels to be the “outer framework” (society at large and health care institutions), the “extended care system” (relatives of the patient and their health professionals’ team members) and the “health-professional-patient-relationship”–all of which make their distinctive impacts on dealing with desire to die. Our empirical findings regarding the complexity of the palliative care situation correspond with results from a recent publication by Hodiamont et al. who put forth a conceptual framework that differentiates between the subsystems “social system”, “team” and “patient” [26]. The authors suggest that understanding and shaping situations and dynamics of individual care situations can be improved by applying the systemic view that is attentive to the interactions between agents of all hierarchical system levels. In our study, health professionals’ uncertainty arises from different levels and leads to ambivalences in their attitudes and convictions surrounding desire to die whereas patient’s ambivalence concerns the coexistence of desire to die and will to live.

### Outer framework of palliative care: Taboos, wording and norms surrounding desire to die

Desire to die in palliative care is being recognized in the research literature and becomes increasingly important in contemporary debates [8, 27–29]. However, desire to die is still partially tabooed in society and clinical practice–not least because death is usually not considered a possible and acceptable outcome of treatment [30]. Sometimes health professionals feel the patient’s death or desire to die is their personal failure or a result of unsuccessful treatment, intensified by a public believe that modern medicine is able to cure all ailments [31].

The ambiguity of vocabulary concerning near death and desire to die and its insecure use on all sides adds to the taboo [32, 33]. This may worsen the quality of care [34]. A change towards more appropriate language may lead towards increased mindfulness and specificity. For example, if the discontinuation of a certain treatment strategy may be adequate, health professionals should not describe it as “withdrawal of care” as that implies turning away from ongoing patient needs [34]. Rather they should pay attention to the maintenance of quality of life, help with unfinished business and support their patients in making the best of their remaining lifetime. Another example is the distinction between ‘palliative sedation’ and so-called ‘terminal sedation’. Whereas palliative sedation is an intervention aiming at reducing therapy-refractory pain, so-called terminal sedation aims at treating terminal restlessness (e.g. in terminal delirium) such that it would be more accurate to speak of ‘therapeutic palliative sedation’ to mark the difference [35]. As for the treatment of terminal restlessness with sedative medication, we recommend not using the (potentially guilt-ridden) wording ‘terminal sedation’ as ‘terminal’ herein might be taken to imply the premature ending of life while this is not what the intervention aims at. Especially in conversation with patients and relatives, it might be better to simply describe the intervention and state its goals.

Patients, their relatives and health professionals are only partially informed about the legal options for dignified care corresponding to the patient´s desire to die and how to communicate these options adequately [35]. A recent literature review has found that the legislative prohibition on health professionals initiating conversations about voluntary assisted dying in Australia (Section 8 of the *Voluntary Assisted Dying Act*) may lead to less optimal patient outcomes (e.g., not being informed about all available treatment options) [36]. Within the German statutory framework, changing treatment plans, withholding and discontinuation of treatment as well as therapeutic palliative sedation can be reasonable and legally permitted measures [37]. Euthanasia and assisted suicide were legally restricted in Germany at the time of data collection [11]. In February 2020, the Federal Court of Justice declared the restriction of assisted suicide to be inadmissible [13]. As taking one’s own life is not a punishable offence in Germany, assisting suicide must also be exempt from punishment. The concrete consequences of this judgement remain to be seen. What is certain, however, is that controversial discussions on the topic will continue. Regardless of the (future) design of national laws, the German Society for Palliative Medicine recommends respecting a patient’s desire to die, bearing with it and speaking openly about it [38]. This requires a taboo-free approach, which does not, however, equal agreeing with the patients’ wishes to end their lives. Rather, talking about it and thereby widening one’s view to possible alternatives can also be considered as suicide prevention [5]. In the context of the latest German case law, it was stressed that we may regret patient decisions in favor of (assisted) suicide and may try everything to change their mind–but in the final consequence, we must accept their free decision. Hence, ultimately access to assisted suicide options must not depend on physicians showing a willingness to support related requests, if they are persistent and serious. The still pending legal regulations in Germany must guarantee availability of suicide prevention measures as well as access to assisted suicide. Importantly, no individual medical practitioner can be forced to provide assistance in suicide [13].

#### Gender effects

In general, no effects of age or profession were found in the panelists’ statements. The identified gender effects were sparse and may be interpreted with caution: more women than men commented on the importance of specific patient needs as well as the inclusion of patient relatives–a perspective focused on social interaction. In contrast, more men than women took an abstract perspective and considered the impact of the surrounding frameworks. One possible explanation might be effects of gender stereotypes regarding agency and communion: empirically, women tend to display more communal traits whereas men tend to display more agentic traits [39]. These manifest in assumptions about how women and men experience and affect the world, which become part of one’s gender identity by socialization processes. Gendered professional styles such as these, including socially focused communication styles that are more often found in women, are not written into stone but are in fact prone the change (for example by communication trainings) [40].

### Extended care system: Co-working with relatives and interdisciplinary teamwork in palliative care

Health professionals are uncertain whether and how to talk about desire to die not only with patients but also with their relatives [41]. The family is in special need in case of a patient’s desire to die [42]. Furthermore, collaborating with relatives can be a potential resource for dealing with desire to die [43] if the patient agrees to involve them. This can produce opportunities, but may also harbor risks. On the one hand, relatives may have an intimate knowledge of the patient’s preferences and needs and can make a significant contribution to improving individual palliative care [44]. On the other hand, relatives may overestimate their knowledge of what the patient values and needs or might have deviating opinions [45]. Health professionals should thus be able to determine when and to what degree the involvement of relatives is adequate [46]. Asking the patient what they want, i.e. how involved they want their relatives to be, should be the first step towards this goal.

In case of a patient’s potential desire to die, multidisciplinary team meetings–also pointed out as important by our Delphi panelists–may enable the combination of complementary expertise and perspectives [42]. Another benefit of collegial exchange may be the reduction of individual team members’ burden [47]. If team members express divergent practical attitudes and ethical stances, convoking ethical case conferences was shown to be helpful in finding solutions and learning to withstand the resulting ambivalences [48].

### Health-professional-patient-relationships: Ambivalences and tensions surrounding desire to die conversations in palliative care

Our Delphi panelists describe the potential tensions between different perspectives of patients and health professionals as particularly challenging. In light of a patient’s death and dying distress, the health professionals’ own needs may fade into the background. At the same time, it is important for health professionals to remain sensitive of their own emotional limits [49]. Stepping back from caring in overwhelming situations does not equal abandoning the patient but can be a responsible and authentically self-reflected decision [37]. If health professionals feel their abilities and competences are inadequate to support a patient with a (potential) desire to die, they should have a network of colleagues whom they can refer these patients to.

#### The self and the others: Health-professional-self-care and sharing patient realities

Patient-centered care, which is respectful of and responsive to individual patient preferences, needs and values, is considered to be an end in itself, not merely a means to achieve other health outcomes. That in mind, communication should be tailored to patients’ needs to permit meaningful deliberation [50, 51]. In relationships with others, we get a chance to share their realities and hence understand them better. The experience of shared reality is a fundamental human need [52] satisfying both the desire to understand and the wish to connect. Shared reality can be established via communication [53]. In the desire to die context, it can provide patients with an access to the health professionals’ expertise, helping them to understand something about death and dying such as a more thorough insight of their expected disease trajectory. Respectively, health professionals can try to take patients’ perspectives in order to address relational needs. It is important to stress that showing understanding for a patient’s desire to die does not equal agreement to hasten death. However, holding their desire to die secret might make patients feel less connected to others and may cause negative health and well-being [54, 55]. Being distant towards others, dissatisfaction with social life and a perceived lack of participation are described as predictive social variables of the wish to hasten death in older adults [56].

#### Desire to die and will to live

Our panelists point out that knowing about the variety and changeability of desire to die and the will to live is also key in providing optimal care. In one of our earlier studies, we have explored this relationship in the context of palliative care [57]. Seemingly paradoxical alternations such as expressing a desire to die while at the same time requesting curative therapies [57, 58] are challenging to health professionals. They can be approached with a nuanced conceptual understanding of desire to die: Living well as long as possible even in the face of suffering and tolerating the possibility of death even while holding onto life go hand in hand [59]. When assessing a patient’s current condition, the possible changeability of desire to die over time has also to be taken into account [60]. Hence, related conversations should be understood as a dynamic, continuous process without a compelling need for immediate intervention. The German palliative care guideline also suggests that in case of persistent desire to die, health professionals should empathically accompany their patients both in and out of conversations thereabout [10].

#### Expressed concerns and potential benefits relating to (proactively) addressing desire to die

Our panelists described tensions between their general knowledge on palliative care and standardized communication guidelines on the one hand and the patient’s individuality on the other hand. Therefore, the clinical approach for (proactively) addressing desire to die in palliative care promotes a patient-centered approach and allows for a great degree of freedom in application due to its semi-standardized structure [17].

The basic intervention recommended first, however dire the patient’s situation may be, is to engage them communicatively [10]. This is based on the assumption that an open discussion on (potential) desire to die in a respectful atmosphere can take the pressure off, illuminate possible backgrounds and open up new perspectives. As such, the recommendation on proactively addressing desire to die included in the semi-structures approach reached an expert consensus agreement of over 80% within the data that the secondary analysis reported here is based on [17]. The secondary analysis showed that there nonetheless exists a particular ambivalence concerning proactively addressing desire to die. While for some patients it is assumed to be relieving and helpful to detect potential for improvement in palliative care, there is also the fear of upsetting others or breaking a taboo. These kinds of arguments against proactively addressing desire to die most often stem from a concern about patients’ welfare and a wish to protect them [37]. Some health professionals worry that patients might interpret questions about possible desire to die as abandonment and that their life may not be considered worth living anymore [61]. Furthermore, not all patients might want to talk about desire to die, their prognosis or impending death. This could be interpreted as denial, even if it may be adequately related to their biographical or cultural background [62]. Although little research exists on the iatrogenic risk of desire to die assessment specifically, meta-analyses and reviews that can cautiously be used as indirect evidence confirm that asking about suicide neither causes nor increases suicidal ideation, behavior and psychological distress [4] and that therefore assessing desire to die probably causes no harm. Importantly, robust effects indicate that asking about suicidality can actually decrease suicidal ideation [4, 63]. Even if psychological distress increases temporarily post-assessment, affected participants still describe those conversations as acceptable and even meaningful [4]. These findings include short-term and long-term post-assessment evaluation and apply to the general public as well as to some vulnerable, high-risk populations [63]. In addition, a recent study found that cancer patients did not experience an ad hoc semi-structured clinical interview for proactive assessment of a wish to hasten death as distressing [64]. They rather considered it important regardless of whether they were personally affected or not.

Beyond keeping up an ongoing conversation with the patient about their desire to die, most best practice recommendations point towards addressing background and functions of it [10]: A strong predictor of desire to die is pain [65], therefore rigorous pain assessment and alleviating symptom burden are mandatory. In general, reasons a person might express for their desire to die can give insight into their acute wishes, needs and fears. Therefore, a desire to die conversation can be a starting point to more fundamental changes in a persons’ care plan, e.g. by deciding for moving into hospice care or adjusting a personal advanced care plan [66, 67]. In this line, open communication about all treatment options, including the possibility of therapeutic palliative sedation, is also recommended as it might reduce fears concerning future unbearable suffering and help in regaining a sense of control. If a desire to die remains persistent, a depression screening is recommended and psychiatric counsel can be helpful [10]. If a patient has a persistent desire to die and asks for assisted suicide but the responsible health professional is not able, willing or eligible to act on this wish, they should nonetheless acknowledge the existence of this wish [5]. Should a health professional decide that they can no longer provide care for their patient who desires to die, they should have a network of referral to whom they can transfer their patient [37].

### Limitations

Our study predominately addresses the context provided by the German legal framework and national differences in legislation pertaining to euthanasia and assisted suicide can go hand in hand with differences in dealing with desire to die. Nevertheless, we believe that our findings hold value for an international audience as regardless of the legal situation, how we deal with desire to die of seriously ill people characterizes our societies.

The presented discussion among health professionals is limited to dealing with verbal expressions of desire to die. Patients who have either lost the ability to communicate verbally as a consequence of their illness (e.g. in late stages of dementia) or do not speak the health professionals’ language may not talk about desire to die. This situation is especially challenging as a lack of communication often means a lack of social connection that can foster feelings of isolation and a low quality of life [68] which might strengthen a desire to die. Even though adaptive interaction with a focus on nonverbal language [69], alternative communication technologies [68] or calling in a (professional) interpreter [70] can alleviate these difficulties to some degree, the question of how to adequately assess or discuss the desire to die of such patients still needs further research.

## Conclusions

Taboos, uncertainties and ambivalences surrounding desire to die occur on all levels of palliative care structures–from the most general societal coordinates to the most specific details of individual relationships. On the level of the ‘outer framework’, we identified (1) the ambiguity of vocabulary surrounding death and dying as well as (2) misinformation about legal options in end of life care as sources of uncertainty and (3) the challenge of adapting a taboo-free approach towards desire to die that does not equal agreeing with a patient’s wish to hasten death as controversial aspects. Within the ‘extended care system’, (4) health professional’s uncertainty in talking about desire to die with the relatives of their patients and (5) disagreement in the team as a source of tension emerged as controversial aspects. On the level of the ‘health-professional-patient-relationship’, we identified as controversial aspects and related challenges: (6) disagreement between patient and health professional as a source of tension, (7) understanding that desire to die must not equal agreement with wish to hasten death, (8) desire to die and will to live can coexist, (9) general knowledge does not cover individual patient needs, resulting in health professional’s uncertainty, (10) ambivalence about proactively addressing desire to die in palliative care and (11) acknowledging the existence of desire to die (including the request for assisted suicide) even when the health professional doesn’t want to deliver assisted suicide.

These aspects influence our convictions on how to address desire to die both in clinical practice and from the theoretical point of view. Hence, dealing with desire to die will remain an ambivalent challenge for all involved that we must take on in the interest of optimal patient care.

Desire to die appears as a factor reworking the relations between health professionals and patients and the degree to which they can partake in each other’s realities. The phenomenon is intertwined with the will to live and while death and life are common themes of all human existence, healthcare routines might not correspond well with a particularly individual case. We then must attune to the patient. A broad conceptualization of desire to die and the capacity to withstand ambivalences can help us in doing so.

Proactively addressing desire to die can be a possible way to open up conversations about desire to die. However, these conversations–especially the proactive approach–are also viewed critically by some practitioners in the study. With this in mind, we nevertheless propose that communication about desire to die should be initiated proactively if realized in an open-ended, empathic manner and guided by a sound clinical rationale such as our semi-structured clinical approach [17]. This recommendation is in line with an expert consensus that grounds the recommendation to proactively address desire to die in the *German Palliative Care guideline for patients with incurable cancer* [10]. Such conversations among health professionals and their patients can be adequate interventions in palliative care. They can reveal ambivalences, taboos and uncertainties surrounding desire to die and even if they cannot all be resolved, they are easier to handle when they are disclosed.

In summary, our five most important recommendations for health professionals treating patients who (potentially) desire to die are:

1. Use of appropriate language and terminology in open communication about desire to die
2. Reflecting taboos and myths surrounding desire to die communications and recognizing (potential) resistance against proactively addressing desire to die
3. In case of desire to die: (re)assessing physical symptom burden and alleviating pain;
4. In case of persistent desire to die: screening for depression and considering psychiatric counsel;
5. In case of persistent and serious requests for assisted suicide: acknowledge the request, deepen the conversation, and ensure the appropriate response in light of the national legislation.

With respect to the outer framework of desire to die, we suggest encouraging public dialogue on a national and international level, and creating spaces for culture-sensitive debates in science, policy, and clinical practice.
